# Prognostic significance of HLA EMR8-5 immunohistochemically analyzed expression in osteosarcoma

**DOI:** 10.1186/1746-1596-9-72

**Published:** 2014-03-25

**Authors:** Ola H Nada, Naglaa S Ahmed, Hoda H Abou Gabal

**Affiliations:** 1 Department of pathology, faculty of Medicine, Ain Shams university, Cairo, Egypt; 2 Department of pathology, faculty of Medicine, Ain Shams university, Cairo, Egypt; 3 Department of pathology, faculty of Medicine, Ain Shams university, Cairo, Egypt

**Keywords:** Osteosarcoma, Human leukocyte antigen, Prognostic factors, Immunohistochemistry

## Abstract

**Background:**

Defects in Human Leukocyte Antigen (HLA) class I antigen expression and/or function in tumor cells have been extensively investigated, because of their potential role in the escape of tumor cells from T cell recognition and destruction. The researchers evaluated HLA class I expression in tumor tissue as a prognostic factor in osteosarcoma patients and as a predictor of their survival. This retrospective cohort study was conducted at the pathology laboratory of Ain Shams University Hospital, and Ain Shams University Specialized Hospital during the period between January 2009 and January 2012.

**Methods:**

The researchers investigated HLA class I expression in primary osteosarcoma by immunohistochemistry using EMR8-5 mAbs. Furthermore, researchers evaluated the correlation between HLA class I expression and the clinicopathological status and outcome in formalin fixed paraffin embedded tissues from thirty six (36) patients with osteosarcoma.

**Results:**

A high expression of HLA class I was detected in 18 (50) % of tumor samples examined; while tumors with low or negative expression represented 9 (25%) cases each. Data indicate that the overall survival rate of patients with tumors highly expressing HLA class I was significantly higher than those with low or negative expression.

**Conclusion:**

Down-regulation of class I antigen expression is associated with features of aggressive disease and a poorer prognosis. Therefore, it is imperative to identify HLA as a prognostic factor at the time of diagnosis to detect chemotherapy-resistant tumors and to generate a modified treatment regimen.

**Virtual slides:**

The virtual slides for this article can be found here: http://www.diagnosticpathology.diagnomx.eu/vs/1159334857109547.

## Introduction

Osteosarcoma (OS) is the most common primary malignant bone tumor in children and adolescents with a high metastatic potential [1,2] comprising approximately 20% of overall bone tumors and about 5% of pediatric tumors [3,4]. OS is the fifth most common malignancy among individuals aged 15 to 19 years, and the second most common in adolescents after lymphoma. The incidence of osteosarcoma in the general population is 2–3 million/year, but is higher in adolescence, in which the annual incidence peaks at 8–11 million/year at 15–19 years of age [5]. Boys are reported to be affected more frequently than girls [6].

Osteosarcoma occurs primarily in the metaphysis of long bones around the knee region of the distal femur or proximal tibia in about 80% of cases. In the remaining 20% of the cases, OS occurs in the axial skeleton and pelvis [5,7]. Commonly affected bones, in descending order, are the femur (40%), the tibia (20%) and humerus (10%) [8]. OS is a highly aggressive neoplasm having a broad spectrum of histologic appearances. Depending on the predominant type of extracellular matrix, conventional osteosarcoma is classified into osteoblastic, chondroblastic and fibroblastic [9]. This separation is largely artificial, because there is no statistical difference in the survival of patients with high-grade tumors among these 3 histologic types. Moreover, the treatment for all types is similar [10].

The 5-year event-free survival rate for patients with localized disease has reached 70% [11], in contrast to 20% for patients with metastatic osteosarcoma [12,13]. Until recently, both types of patients have been treated using the same therapy according to the discretion of physicians. This signifies that these patients do not usually receive consistent and homogenous treatment. Fifty percent of patients with osteosarcoma of the extremity can be cured with a relatively nonaggressive regimen of chemotherapy [14]. One-third of patients with localized osteosarcoma experience recurrent or progressive disease [15,16] and the average survival period after a recurrence is <1 year [17]. Therefore, to improve the survival rate of osteosarcoma patients and to improve their overall wellbeing, it is imperative to develop continuously novel therapeutic strategies and prognostic markers to define different risk groups [18].

A number of clinical and pathologic features such as clinical stage have been reported to be correlated with survival [19] in addition to patient’s age and gender [20], tumor site [21], type of surgery [22], and local recurrence. The histologic response of the tumor, identified by the degree of necrosis, to the preoperative chemotherapy is currently the most sensitive indicator of survival [21]. Moreover, serological and molecular markers, such as alkaline phosphatase and lactate dehydrogenase, p-glycoprotein, cxcr4, survivin, and ezrin, are powerful predictors of survival for patients with osteosarcoma [23]. Nonetheless, individually, these prognostic factors have limited utility in predicting survival [24]. Recently, research on osteosarcoma has focused on identifying novel therapeutic targets and prognostic markers. Several molecular targets are currently under evaluation for osteosarcoma. There is, however, insufficient data to allow any of these targets to be recommended as prognostic factors or therapeutic targets [25].

Recently, immunotherapeutic trials have been suggested and conducted for bone tumors; including human leukocyte antigen (HLA) class I antigens. HLA class I antigens are trans-membrane glycoproteins expressed on the surface of most nucleated cells in the human body [26]. They have a central role in the cell-mediated immune system. Cytotoxic T lymphocytes (CTLs) can recognize antigenic peptides presented on the cell surface with HLA class I molecules, and kill the target cell, thus allowing the destruction of tumor cells by the immune system [27]. Down-regulation of HLA class I was found to be implicated in the immune escape of malignant tumors [28]. In addition, the frequency of HLA class I defects is reportedly higher in metastatic lesions than in primary or premalignant lesions [26]. Independent of the traditional prognostic markers for these cancers, HLA class I antigen down-regulation as a biomarker has also been shown to have prognostic powers in breast, esophageal cancer [29], malignant melanoma [30], lung [26], ovarian cancers [31], colon cancer [32] and renal cell carcinoma [33]. As such, it affects survival of patients with these diseases [27]. The loss of HLA class I molecules has been discussed in the context of tumor aggressiveness, such as differentiation of histology, invasiveness, and metastatic potential [34,35].

The major goals of cancer biology studies are to identify prognostic factors and therapeutic targets. Gene and protein expression array data may soon provide customized information on tumor prognosis and metastatic potential, as well as indications of possible tumor targets for selective therapy. The objective of this study is to evaluate HLA class I EMR8-5 as a potential prognostic factor in osteosarcoma patients at time of diagnosis that is capable of predicting their survival.

## Material and methods

This is a retrospective cohort study, which includes formalin fixed paraffin embedded tissue blocks from 36 patients with osteosarcoma, for whom medical records were archived in the pathology laboratory of Ain Shams University Hospital, and Ain Shams University Specialized Hospital during the period between January 2009 and January 2012. This study obtained the institutional review board approval from Research Ethical Committee at Faculty of Medicine, Ain Shams University. Written informed consent was obtained from the patients for the publication of this report and any accompanying images.

Inclusion criteria for selecting the tumor tissue blocks are as follows: (1) Tumor is a primary osteosarcoma located in the extremity; (2) Age of the Patients is under 40, having received chemotherapy and limb salvage; (3) Archival paraffin embedded tissue of the pretreatment diagnostic biopsy and the resection specimen are available; (4) Resectable metastatic nodules in non-localized osteosarcoma; and (5) Clinical follow up data after initial diagnosis is available.

Clinic-pathologic variables were recorded and included age, gender, tumor location, maximal tumor diameter on initial magnetic resonance images, histologic subtype and status of stage (localized versus metastasis) at time of diagnosis. Also, laboratory results for serum alkaline phosphatase at presentation and follow up data, starting from the time of initial biopsy until the death of the patients, disease occurrence in any site (distant metastases or local recurrence) or the last follow up were also recorded. Survival indicators (the overall survival and disease free survival) were identified from the follow up data.

Paraffin embedded tissue blocks of the pretreatment diagnostic biopsy and the post treatment primary resection specimens of 36 osteosarcoma cases were cut at 4 microns thick sections and subjected to the following examinations:

### I. Routine haematoxylin and eosin staining

H & E stained slides of the initial pretreatment biopsies were examined and revised to evaluate the histopathologic type and tumor grade, which is based on the recent WHO criteria for histopathologic classification*.* The post-treatment primary resection specimens were examined for the evaluation of the histologic response of the preoperative chemotherapy. The results were considered good if the extent of tumor necrosis was ≥ 90% and poor if it was < 90%*.*

### II. Immunohistochemical staining for HLA EMR8-5 for sections of the pretreatment diagnostic biopsies

Tissue sections were deparaffinized, rehydrated then endogenous peroxidase activity was quenched by 10 min incubation in 3% hydrogen peroxide in methanol. After antigen retrieval and protein block, the primary antibody [Mouse monoclonal [EMR8-5] to HLA Class 1 ABC, diluted by PBS, 1:100, Abcam, Catalog No. ab70328] was applied using Sequenza center for immunostaining. The secondary antibody (supersensitive immunodetection system (Biogenex, catalog No: AD000-SL) was then applied followed by peroxidase labeled streptavidin. Slides were incubated for 10 minutes with substrate chromogen (DAB) mixture. Finally, the slides were counterstained and mounted with Canada balsam. Colon carcinoma tissue sections were used as positive control. Both positive and negative control slides were included in each run.

All specimens were reviewed independently using light microscopy for at least five areas at a 400× magnification by the investigators, blinded with respect to the immunohistologic and clinical data. The reactivity of HLA class I EMR8-5 was determined by staining of the plasma membranes of tumor cells. The expression status of HLA class I was graded semiquantitatively according to the following modified classification: negative (positive cells < 5%), low (≤ 5% positive cells ≤ 50%), and high (positive cells > 50%). Diffuse expression and heterogeneous expression were regarded as high grade and low grade, respectively. Focal expression was graded as low or negative according to the percentage of positive cells [36].

#### Statistical analysis

The data collected was revised, coded, tabulated and introduced to a PC using Statistical Package for Social Science (SPSS 15.0.1 for windows; SPSS Inc, Chicago, IL, 2001). Continuous variables are expressed as mean and SD. Categorical variables are expressed as frequencies and percentages. Student T Test was used to assess the statistical significance of the difference between two study group means. ANOVA Test was used to assess the statistical significance of the difference between more than two study groups mean. Fisher’s exact test was used to examine the relationship between Categorical variables when the expected count is less than 5 in more than 20% of cells. The endpoints of this study were both disease-free survival and overall survival. Disease-free survival is defined as the period as of the date of initial biopsy until disease occurrence (distant metastases or local recurrences) or last follow up. Overall survival is defined as the period lapsing from the date of initial biopsy until death or last follow. Patients who remained alive at the time of data cut-off were censored at the last date the patient was known to be alive. Survival distributions were estimated using the Kaplan–Meier method. Correlations between survival and potential prognostic features were analyzed using the log-rank test. Cox Regression was used for modeling the time to a specified event, taking into consideration the values of other given variables. Univariate and multivariable analyses of the postoperative outcome were conducted using Cox’s proportional hazards model. P- Value (level of significance) < 0.05 is designed as Significant (S) while *P = .*01 denotes highly significant (HS).

## Results

### Results of the clinicopathological data collected

Among the 36 studied cases, 27 (75%) were male and 9 (25%) were female, at a proportion of 3:1. The age ranges from 12 to 28 years, with a mean of 15.42 ± 4.17 years. Eighteen patient (50%) were ≤14 years and 18 patients (50%) were >14 years. Regarding the anatomical location of the tumor, 27 (75%) tumors were in the femur, particularly in its distal third 21 (58.3%), while the remaining nine tumors were located in the tibia (25%). The size of these tumors ranged from 8.5 to 20 cm with a mean of 11.82 ± 4.54. Preoperative computed tomography demonstrated metastasis in nine patients (25%).

### Results of the examination of haematoxylin and Eosin tissue sections

Evaluation of the histopathological sections revealed that osteosarcoma variants were subdivided into 12 (33.3%) chondroblastic, 9 (25%) fibroblastic, 12 (33.3%) osteoblastic and 3 (8.3%) small-cell tumors. By examination of postoperative tissue sections from primary resection specimens for tumor necrosis, researchers found that 24 (66.7%) responded poorly to preoperative chemotherapy, whereas only 12 (33.3%) of the tumors responded well.

### Results of the expression of HLA class I in osteosarcoma

Immunohistochemical staining was evaluated and out of the 36 osteosarcoma specimens, 18 (50%) were graded as having high expression of HLA class I molecules; 9 specimens (25%) were graded as having low expression; and the remaining 9 specimens (25%) were negative for HLA class I expression. Collectively, the expression of HLA class I was lost (negative-grade expression) or down regulated (low-grade expression) in 50% of the tumor tissues.

There were significant statistical differences in the distribution of immunohistochemical scores of HLA class I among the different tumor stages at the time of presentation (localized stage versus metastases stage) (P = .0001). Negative HLA class I was observed significantly more often, in cases with metastatic disease compared to cases without metastases. None of the metastatic cases at time of diagnosis showed high HLA class I expression (Table 1). A highly significant relationship was also found between HLA class I expression and the sex of the patients (P = .000); all cases showing high HLA class I expression were males (Table 1). Similarly a highly significant association was noted between HLA class I expression and histopathological variants (P = .0001). Nonetheless, the relationships of the other clinicopathological parameters including age, tumor site, serum ALP at time of presentation, extent of tumor necrosis were statistically insignificant (Table 1).

**Table 1:** Comparison of the clinical characteristics of studied 36 cases with HLA class I expression

		**HLA class I expression**						**P**	**Sig**
		**Negative**		**Low**		**High**			
		**N**	**%**	**N**	**%**	**N**	**%**		
**Age group**	≤14 years	3	33.3%	6	66.7%	9	50.0%	.473*	NS
	>14 years	6	66.7%	3	33.3%	9	50.0%		
**Sex**	Male	6	66.7%	3	33.3%	18	100.0%	.0001*	HS
	Female	3	33.3%	6	66.7%	0	.0%		
**Tumor stage at diagnosis**	Localized	3	33.3%	6	66.7%	18	100.0%	.0001*	HS
	Metastatic	6	66.7%	3	33.3%	0	.0%		
**Tumor site**	Lower femur	3	33.3%	6	66.7%	12	66.7%	.250*	NS
	Middle femur	3	33.3%	0	.0%	3	16.7%		
	Upper tibia	3	33.3%	3	33.3%	3	16.7%		
**Tumor necrosis**	Poor response	8	88.9%	6	66.7%	10	55.6%	.258*	NS
	Good response	1	11.1%	3	33.3%	8	44.4%		
**Histopathological variant**	Chondroblastic	3	33.3%	6	66.7%	3	16.7%	.0001*	HS
	Fibroblastic	0	.0%	0	.0%	9	50.0%		
	Osteoblastic	6	66.7%	0	.0%	6	33.3%		
	Small cell	0	.0%	3	33.3%	0	.0%		
**Serum alkaline phosphatase**	Mean ± SD	592.1	372.6	482.5	298.9	324.0	228.8	.078**	NS

*Fisher exact.

**ANOVA.

### HLA class I expression relating to survival in primary osteosarcoma

To investigate the relationship between HLA class I expression and the clinical outcome, researchers analyzed patient survival relating to HLA class I expression in the primary tumor. The duration of follow-up for the entire study group ranged from12 to 50 months with the mean of 29.8 ± 7.3 months and median of 30 months, during which 8 patients (22.2%) died of disease; length of survival ranged from 12–36 months with mean of 24.6 ± 8.1 months and median of 27 months. Twenty-three patients (63.8%) developed a disease (relapse), either metastatic disease or local recurrences; interval to disease recurrence ranged from 2–40 months with a mean of 12.1 ± 9 months and median of 10 months. The overall survival rate at 1, 2 and 3 year was 97.2%, 91.7% and 65.5% respectively; with a mean of 42.4 ± 2.4 months. However, the disease-free survival at 1, 2 and 3 year was 58%,41.7% and 38.9% respectively; with a mean of 24.3 ± 3 months and a median of 18 months.

**Figure 1:**
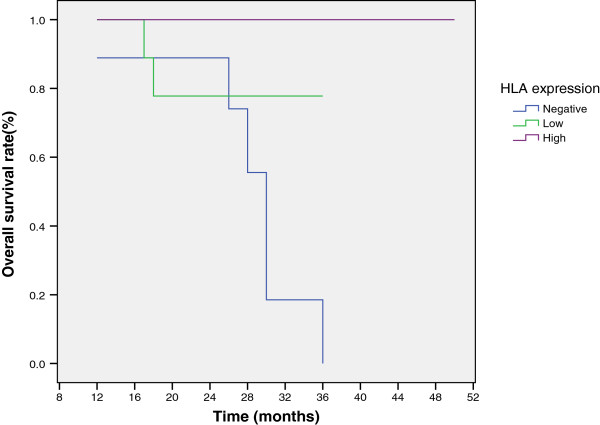
Kaplan-Meier overall survival curve in relation to immunohistochemistry scores for HLA class I.

**Figure 2:**
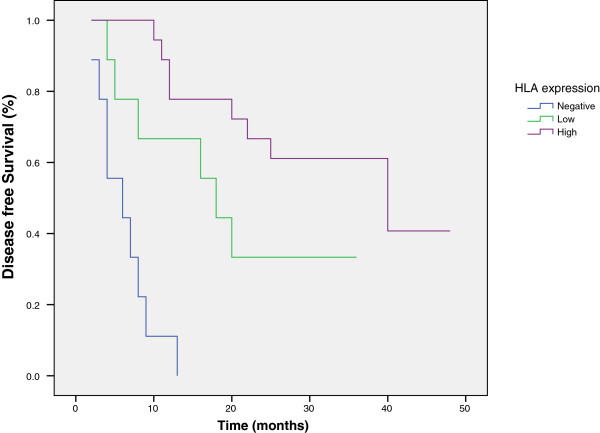
Kaplan-Meier disease free survival curve in relation to immunohistochemistry scores for HLA class I.

Through the use of univariate analysis of the overall and the disease free survival in relation to the HLA class I expression (Table 2), Log Rank tests performed on Kaplan – Meier survival curves disclosed a highly significant differences in overall and disease free survival (P = .0001 for both) (Figures 1 and 2). Patients who had high expression of HLA class I in their initial biopsies showed a significant better overall survival compared to those with low expression of HLA class I, or those with negative expression. As regards the disease free survival, patients with osteosarcoma highly expressing HLA class I had a significantly better disease-free survival compared to those with negative expression. Similarly, osteosarcomas with low expression of HLA class I showed a significantly longer disease-free survival when compared to those with negative expression. When excluding patients presented with metastatic disease, there were still a significant differences between HLA class I expression, on the one hand, and overall (P = .018) and disease free survival (P = .001), on the other.

**Table 2:** Univariate survival analysis of osteosarcoma according to immunohistochemistry scores for HLA class I and other clinicopathologic factors

	**3 year overall survival rate**	**3 year disease free survival rate**
**HLA class I**	P < 0.0001	P < 0.0001
Negative	0%	0%
Low	77.8%	33.3%
High	100%	66.1%
**Age**	P = 0.93	P = 0.89
≤ 14 years	83.3%	33.3%
> 14 years	60.4%	44.4%
**Sex**	P = 0.023	P = 0.001
Male	80.9%	51.9%
Female	0%	0%
**Tumor site**	P = 0.60	P = 0.081
Lower femur	85.7%	38.1%
Middle femur	0%	0%
Upper tibia	58.3%	66.7%
**Tumor stage**	P < 0.0001	P < 0.0001
Localized	96.3%	51.9%
Metastatic	0%	0%
**Histopathological variant**	P = 0.13	P = 0.413
Osteoblastic	39.3%	50%
Chondroblastic	37.5%	25%
Fibroblastic	100%	55.6%
Small cell type	100%	0%
**Tumor necrosis**	P = 0.017	P < 0.0001
Poor response (<90%)	32.7%	12.5%
Good response (≥90%)	100%	91.7%

In univariate analysis of overall and disease-free survival with clinicopathological parameters (Table 2), the only parameters that reached a statistical significance for overall and disease-free survival were the sex of the patients (P = .02 and P = .001 respectively), tumor stage at time of diagnosis, (P = .0001 for both) and extent of tumor necrosis (P = .017 and P = .0001 respectively). Among female patients included in this study, poor response to preoperative chemotherapy together with metastatic stage at time of diagnosis were associated significantly with shorter overall and disease-free survival when compared to male patients, localized stage at presentation and good response to preoperative chemotherapy.

**Figure 3:**
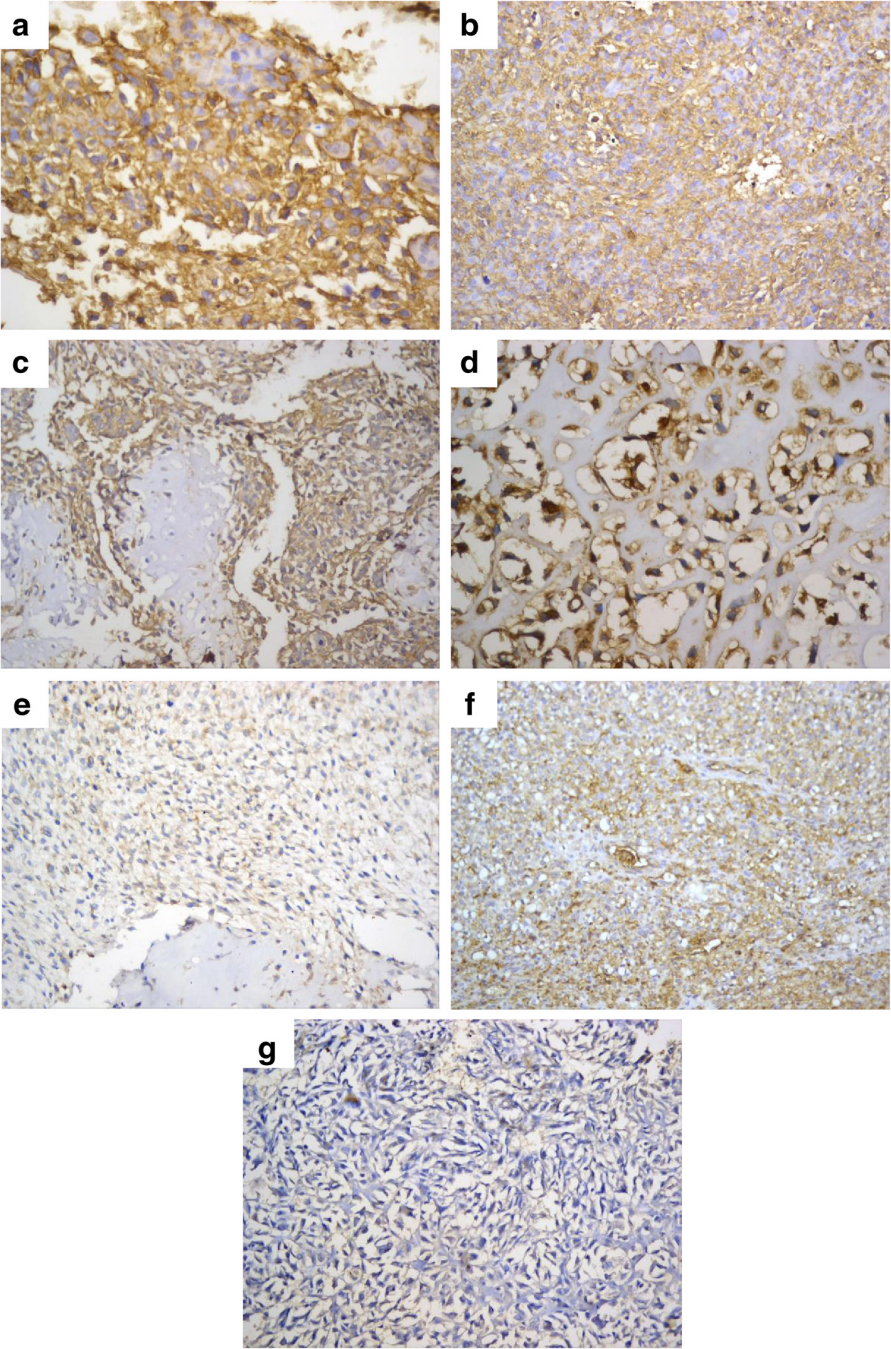
**Representative images of immunohistochemical staining of primary osteosarcoma with HLA Class I EMR8-5 Sections:a, b, candd:** Sections from cases showing strongly positive expression of HLA class I with diffuse staining in > 50% of tumor cells in a conventional osteosarcoma (a, b: immonoperoxidase x 400 and x200 respectively) and chondroblastic osteosarcoma with well evident membranous immunostaining of malignant cells (c, d: immonoperoxidase x400). **e** and **f**: Sections from cases with low positive and heterogeneous expression of HLA class I, respectively, showed areas with both strongly positive and negative expression of HLA class I (immonoperoxidase x200). **g**: Sections from a case with negative expression of HLA class I (immonoperoxidase × 200).

Using the backward stepwise Cox regression analysis, multivariate analyses were performed to determine the variables that were independently predictive of overall survival. As illustrated in Table 3 with adjustment of sex, tumor necrosis and HLA class I expression, metastatic stage at time of diagnosis was the only independent prognostic factor for decreased overall survival that reached a significant level (Hazard ratio = 30.086, P = .002). However, the other variables lost their significance at multivariate analysis. Subsequent Cox regression analysis for disease-free survival (Table 3) confirmed that poor response to preoperative chemotherapy (Hazard ratio = 19.031, P = .007) and negative HLA class I expression (Hazard ratio = 19.125, P = .001) were independent predictors for decreased disease-free survival. Variable HLA expression is illustrated in Figure 3.

**Table 3:** Multivariate analysis for studying the effect of independent factors on overall survival and disease free survival

	**HR**	**P**	**Sig**	**95% CI of HR**	
				**Lower**	**Upper**
**Overall survival**					
Tumor stage at time of diagnosis (localized versus metastatic stage)	30.086	0.002	HS	3.665	246.990
**Disease free survival**					
Female sex versus male sex	1.611	0.538	NS	0.353	7.351
Localized stage at time of diagnosis versus metastatic stage	3.220	0.087	NS	0.845	12.263
Tumor necrosis (good response versus poor response)	19.031	0.007	HS	2.274	159.236
Low HLA class I expression versus high	1.475	0.673	NS	0.243	8.971
Negative HLA class I expression versus high	19.125	0.001	HS	3.359	108.906

## Discussion

Evasion of anti-tumor immunity has been thought to be critical to progress of cancers. Several studies have shown that down-regulation of HLA class I expression is observed in various tumor cells and it would cause a malignant phenotype. It is possible that, in the process of tumor extension, tumors that lost HLA class I survived and escaped from antigen-specific CTL-mediated lysis leading to tumor dissemination and metastasis. Patients with positive HLA class I expression showed a better DFS (disease free survival) in comparison with those with down regulation of HLA class I expression. Our result differs from some studies of esophageal cancer [34] and malignant melanoma [37] that have shown a lack of prognostic significance for HLA class I expression. Total loss of HLA class I was proposed as an indicator of good prognosis in breast cancer and non-small-cell lung cancer [38]. In those reports, the authors mentioned that HLA class I antigen down-regulation may make the tumors more susceptible to natural killer (NK) cell killing and result in a better prognosis. The results of the current study suggest that HLA class I expression may be one of the important prognostic factors in osteosarcoma and an important factor to select proper patients for treatment with CTL-based immunotherapy in the future.

It has been demonstrated that human tumors with various histology have low or down regulated HLA class I molecules due to the modulation or inhibition of the expression of various HLA class I antigen-processing machinery (APM) components. It is well known that abnormality of HLA class I molecules and APM in tumor cells is one of the major reasons for escape from CD8(+) cytotoxic T cells, resulting in disease progression [39]. Moreover, tumor cells showing the down regulation of specific HLA class I alleles could escape from T-cell-mediated immunity and also avoid NK cell-mediated killing due to sufficient HLA class I expression [40].

The monoclonal antibody EMR8-5 can recognize all of HLA A, B, and C heavy chain even in formalin-fixed tissue. In this context, EMR8-5 can recognize whole HLA molecules, and its validity was supported by the immunostaining performed in our study. Some authors reported that HLA class I positivity examined using the conventional HLA class I antibody W6/32, which also recognizes all HLA class I antigens, was 30% of HLA class I positivity in breast cancer, similar to that shown in our present study [38,41]. In contrast, Madjd et al. investigated HLA class I expression in breast cancer using a HC-10 antibody [38], and demonstrated that HLA class I negativity correlated with a better postoperative outcome. These results conflicted with the data in our study. This discrepancy may be explained by the fact that whereas the HC10 mAb scarcely reacts with HLA-A alleles, the anti- HLA class I heavy chain mAb EMR8-5 can detect all recombinant proteins of HLA-A, B, and C alleles [42]. In addition, EMR8-5 can be applied to paraffin-fixed specimens, so in this context it is an ideal antibody for evaluating cancerous HLA class I antigen expression.

In the present study, down-regulation of HLA class I expression represented an independent factor associated with poor prognosis in pretreatment biopsy of non-localized osteosarcoma. The down regulation of HLA class I expression was significantly associated with lymphatic and nodal invasion in studies conducted by Kaneko and his colleagues [35]. Therefore, cancerous HLA class I down regulation seems to be conducive to metastasis to other organs. Other studies reported the down regulation of HLA class I expression in breast cancer [43], esophageal cancer [34], and lung cancer [26]. The degree of HLA class I loss may be affected by organ specificity. Watson et al. (2006) reported that patients who had colon cancer with low-level expression of HLA class I displayed significantly worse prognosis compared with those with HLA class I-positive cancer consistent with the results of the current study [32]. The only research work, in addition to this research, that performed osteosarcoma tissue blocks by immunohistochemistry is in agreement consistently with our findings as the researchers declared that patients with osteosarcoma exhibiting high expression of HLA class I showed significantly better overall and event-free survival compared to those with HLA class I-negative osteosarcoma [44].

Another recent study showed that osteosarcoma tissues with high expression of SOX9, a developmental transcription factor, tend to have shorter overall survival and disease-free survival [45]. Combining SOX9 and HLA EMR8-5 in a large scale study can probably prove their high prognostic value in osteosarcoma. Another promising marker is Aurora Kinase A and B that has proven to be correlated with poor outcome in patient with chondrosarcoma [46]. However, the only research applied for Aurora Kinase A and B on osteosarcoma was in vitro, thus further investigation may evolve a new prognostic marker.

Multivariate analysis showed a significant adverse effect on prognosis for metastatic tumors at time of initial diagnosis. Regarding the prognostic significance of age, a better prognosis for younger patients has been reported [47], whereas other authors found a better prognosis for older patients [18]. Prognostic significance of alkaline phosphatase in osteosarcoma was previously documented [20]. Furthermore, tumors of distal upper limb are associated with better survival [24], whereas tumors in proximal femur or humerus have been reported to have poorer prognoses than those on the sides of knee joints [21]. In our cohort, no significant results were obtained in correlation with age, alkaline phosphatase, or tumor location. Nonetheless, due to different criteria of inclusion, a reliable comparison between studies is impossible.

As the down regulation of HLA class I expression frequently occurred in OS, the preservation of HLA class I expression on tumors might be one of the inclusion criteria for cancer vaccination therapy for OS. Furthermore, the treatment strategy aiming at restoring HLA class I expression could be able to improve survival among patients with OS or could lead to successful immunotherapy.

## Conclusion

The status of HLA class I expression affected the overall survival and disease-free survival of patients with osteosarcoma. Down-regulation of HLA class I expression in osteosarcoma represents a marker of poor prognosis, and may play a critical role in immune surveillance of patients with osteosarcoma. The present results provide critical information for successful immunotherapy against osteosarcoma. However, because of the limited number of cases in our work which are compiled from a single institute, larger studies with multi-institutional collaboration are recommended to validate the significance of HLA class I expression as a prognostic factor and the rational application of immunotherapy for patients with osteosarcoma.

## Abbreviations

OS: Osteosarcoma; HLA: Human leukocyte antigen; CTLs: Cytotoxic T lymphocytes; DFS: Disease free survival.

## Competing interests

The authors declared that they have no competing interests.

## Authors' contributions

ON designed the study, wrote the protocol, monitored the immunohistochemical staining and wrote the first draft of the manuscript. NA and HAG performed the statistical analysis and managed the analyses of the study. All authors scored the immunostaining, read and approved the final manuscript.
